# Management of intractable bladder neck strictures following radical prostatectomy using the Memokath^®^045 stent

**DOI:** 10.1007/s11701-019-01035-9

**Published:** 2019-10-15

**Authors:** A. Nathan, G. Mazzon, N. Pavan, R. De Groote, A. Sridhar, S. Nathan

**Affiliations:** University College London Hospitals, London, UK

**Keywords:** Prostatectomy, RARP, Vesicourethral anastomotic stenosis, VUAS, Memokath

## Abstract

The incidence of vesicourethral anastomotic stenosis (VUAS) post radical prostatectomy varies from 1 to 26%. Current treatment can be challenging and includes a variety of different procedures. These range from endoscopic dilations to bladder neck reconstruction to urinary diversion. We investigated a 2-stage endoscopic treatment, using the thermo-expandable Memokath^®^045 bladder neck stent to manage patients with VUAS post radical prostatectomy. We retrospectively reviewed 30 patients, between 2013 and 2017, who underwent a Memokath^®^045 stent insertion following failed primary treatment (dilation and clean intermittent catheterisation) for VUAS. The mean interval time between prostatectomy and Memokath^®^045 stent insertion was 13 months. The mean follow-up time was 3.6 years with all patients having a minimum of 12-month follow-up. All patients had two previous attempts at endoscopic dilatation with or without incision and a trial of clean intermittent catheterisation. During stage 1, the anastomotic stricture is dilated/incised to diameter of 30 Fr, the stricture length is measured, and a catheter is left in situ. One to 2 weeks later, post haemostasis and healing, an appropriately sized Memokath^®^045 stent is inserted. The stent is then removed 1-year post-op. Our series of patients had a median age of 62 (54–72). Most patients (26) had a robot-assisted radical prostatectomy (RARP) or salvage procedure. Results showed improvement in IPSS scores, IPSS quality of life scores, *Q*_max_ and PVR after the Memokath^®^045 stent was removed compared to pre-operation. With a minimum of 12 months post stent removal, 93% of patients were fully continent, whilst 7% of patients were socially continent. 2 (7%) patients had their stents removed and not replaced due to re-stricturing and stone formation. However, no urinary tract infections, stricture recurrence or urinary retention was observed in the rest of the cohort (93%). Overall, the Memokath^®^045 stent was successful in treating 93% of our patients with VUAS. Our series had minimal complications that were managed with conservative measures and in three patients’ re-operation was needed. In conclusion, the Memokath^®^045 stent is a minimally invasive technique with faster recovery time compared to other techniques such as bladder neck reconstruction or urinary diversion. Additionally, it provides superior patency results compared to other techniques such as bladder neck incision and injection of Mitomycin C. Therefore, this management option should be considered in the management of VUAS.

## Introduction

### Background

Vesicourethral anastomotic stenosis (VUAS) is an uncommon complication post radical prostatectomy. However, in intractable cases, urinary retention and possible management options can be problematic for both surgeons and patients. Current literature estimates the incidence of VUAS to be between 1 and 26% [1–4]. This wide range in incidence is likely due to the evolution of prostatectomy technique from open surgery to robot assisted. In 2007, the CaPSURE study of 3310 men found an incidence of VUAS in 8.4% post radical prostatectomy (RP) [1]. More recent studies of VUAS post robot-assisted radical prostatectomy (RARP) have shown a risk of VUAS of 1.4% [2]. However, salvage robot-assisted radical prostatectomy (sRARP) post radiotherapy or brachytherapy has shown an incidence rate between 22 and 26% [3, 4].

The goals of VUAS treatment include resolving urinary retention, maintaining continence and achieving good physiological voiding. First-line treatment is endoscopic dilation, internal urethrotomy or transurethral resection of the stricture area. Depending on the number of attempts of these treatments, patency rates can vary from 17 to 89% [5–7]. In the case of intractable VUAS, treatment options include injection of mitomycin C with dilation resulting in patency rates of 79% for the first procedure and 86% for a secondary procedure [8]. Further treatment options include bladder neck reconstruction, with patency rates of 60–80% [9] or urinary diversion options such as long-term urethral or suprapubic catheters, vesicostomy or ileal conduit formation. Whilst surgical reconstruction and urinary diversion are associated with better patency rates compared to endoscopic treatments, complication rates, erectile function, continence and patient satisfaction are worse [10, 11].

### Aims

With the aim of providing superior patency, safety and quality outcomes, we retrospectively reviewed 30 patients managed with the nickel–titanium, thermo-expandable Memokath^®^045 stent as an alternative treatment approach to manage intractable VUAS.

### Hypothesis

The use of the Memokath^®^045 stent in patients with VUAS will provide superior quality of life, flow and incontinence outcomes than current treatment options.

## Materials and methods

### Patients

We retrospectively reviewed 30 patients, with an age range of 50–73. They all had a Memokath^®^045 stent insertion following two previous failed endoscopic dilations following VUAS. The median time from RP to Memokath^®^045 insertion was 13 months. All procedures were performed at two sites (University College London Hospital and The London Clinic), by two surgeons (SN and GM).

### Memokath^®^045 stent

The stent is a spiral thermo-expandable nickel–titanium alloy shaped like an hour-glass. The structure is part flexible and part rigid, the result is a thermosensitive ‘shape memory’. The advantage is that a deformed piece of alloy can be restored to its original shape. The alloy softens at temperatures below 7–13 °C and returns to a preformed shape when warmed to a temperature above 55 °C. Furthermore, these stents have a tight spiral structure, which prevents urothelial in-growth between the coils. Its spiral construction allows the Memokath^®^045 stent to conform and adapt to the natural curves of the urinary tract. The resulting lack of outward pressure minimizes the risk of secondary ischemic injury to the urothelium. Furthermore, its titanium component resists corrosion in the urinary tract. The internal diameter of the stent is 20 French (Fr) following delivery [12]. The aforementioned characteristics render this product adequate in the treatment of VUAS.

### Surgical procedure

The endoscopic insertion of the Memokath^®^045 stent was undertaken in 2 stages.

#### Stage 1—urethral dilation

The patient was placed in lithotomy position and a rigid cystoscopy was performed with a 22-Fr cystoscope. Visualization of the Ureteric Orifices (UO) was required. If the UO is very close to the bladder neck, the insertion of the Memokath^®^045 stent is contraindicated. The urethra was examined, and the stricture was dilated or incised to a minimum diameter of 30 Fr to allow for the delivery of the Memokath^®^045 stent. Dilation was carried out via ‘S’-shaped dilators. In difficult strictures, incision and vaporisation of the bladder neck scar tissue was required with a roller ball. Haemostasis is carefully achieved. The stricture length was measured accurately using the graduations on the cystoscope to ascertain the required stent length. Finally, a 22-Fr urethral catheter was left in situ and the patient was discharged.

#### Stage 2—Memokath^®^045 stent insertion

After 2 weeks, when the pain, inflammation and bleeding have settled, the second procedure is undertaken. A repeat cystoscopy was performed and the exact stricture length re-measured. The required Memokath^®^045 stent with the best fit is chosen (stents are available in 5, 10, 20 mm lengths). The protective cover of the Memokath^®^045 stent was removed and the mandrel inside the sheath was replaced by a 30° scope. Once the Memokath^®^045 stent was positioned at the exact stricture site, warm 70 °C sterile water was infused through the proximal connector on the collar of the sheath, causing the expansion of the proximal end of the stent. Further, warm water is infused through the distal connector which expands the distal end. Infusion is continued in both connectors and the stent is visualized by fluoroscopy. When the entire stent is expanded, the scope with the inner sheath is withdrawn under vision holding the outer sheath to prevent stent migration.

Most patients were discharged on the same day of the procedure. Intra-operative gentamicin was given. Patients received a three-day prophylactic antibiotic regime, along with analgesia as required.

#### Memokath^®^045 stent removal

12 months after insertion, the Memokath^®^045 stent was removed. The Memokath^®^045 stent was irrigated via cold sterile water at a temperature of 10–15 °C using a 22-Fr cystoscope. The cold water shrinks the Memokath^®^045 stent and it was removed with biopsy forceps. The bladder was re-scoped to ensure that the patient had a patent and wide bladder neck and to look for stones. The patients were discharged on the same day with a three-day post-procedure course of prophylactic antibiotics.

### Follow-up assessment and data collection

Perioperative data such as operation time and Memokath^®^045 stent length were recorded.

Patients were followed up 4–6 weeks post-procedure, then every 6 months for the first year, and then once yearly. Post-procedure assessment included International Prostate Symptom Score (IPSS), quality of life (QoL), incontinence (number of pads), flow rates and post-voiding residuals. Complications and Memokath^®^045 stent failure or removal were also recorded.

### Statistical analysis

Statistical analysis was performed using SPSS 15.0 and microsoft excel. Results for continuous variables were described as Mean and Standard Deviation, whilst categorical variables were described as the number of patients and percentages.

## Results

### Pre-operative results

30 patients were reviewed in this data set, with a median age of 62 and an inter-quartile range of 54–72 (Table 7). 24 (80%) patients had primary prostatectomy, of which 16 (53%) was robot assisted; 3 (10%) was laparoscopic, 1 (3%) was open and 4 (13%) had adjuvant radiotherapy. 6 (20%) patients had sRARP (Table 2). All 30 patients had received two attempts at endoscopic dilation and clean intermittent catheterisation. The mean time between prostatectomy surgery and Memokath^®^045 Stent insertion was 13 months (Table 3). 20 (67%) patients were catheter dependent for voiding, whilst 10 (33%) were catheter independent (Table 4) but with a significantly obstructed voiding pattern of *Q*_max_ 6 mls/s (± 2) and post void residual (PVR) of 176 mls (± 52) (Table 7).

### Intra-operative results

In 26 (87%) patients, the 5-mm Memokath^®^045 stent was used, 3 (10%) required 10 mm and 1 (3%) required 20 mm who had previous brachytherapy and anastomotic displacement (Table 5). The mean operation time for stage 1 was 22 (± 6) mins and for stage 2 was 12 (± 4) mins. All patients went home the same day (Table 6).

### Post-operative results

Median time of follow-up was 3.6 years (2.2–4.8), with all patients completing a minimum of 1-year follow-up.

The results show an improvement in International Prostate Symptom Score (IPSS) from an average of 28 pre-operation to 10, 12 months after the Memokath^®^045 stent removal. IPSS Quality of Life score also improved from 5 (unhappy with symptoms) to 2 (mostly satisfied with symptoms). The *Q*_max_ and PVR baseline was measured only for the ten patients who were catheter independent pre-operation. *Q*_max_ showed improvement from 6 to 14 mls/s and PVR improved from 176 mls to a negligible 22 mls (Table 7).

Importantly, incontinence results showed at short-term follow-up of 3 months and medium-term follow-up of at least 12 months post removal, 93% of patients were fully continent with 100% of patients being fully or socially continent (Table 7).

Note: In two patients, there was Memokath^®^045 stent removal due to failure; therefore, only 28 patients had the full follow-up.

### Complications and failure

2 (7%) patients suffered from dysuria, 2 (7%) patients had ejaculatory pain and 2 (7%) patients had temporary urge incontinence, they were all managed conservatively to good effect. In 2 (7%) patients, the Memokath^®^045 size was too long and overlapped the sphincter; so, the stents were replaced. In 1 (3%) patient, the stent migrated due to inappropriate urethral catheterisation and it was replaced operatively (Table 8).

In 2 (7%) of patients, the stent failed and was removed but not replaced; one due to re-stricture through the stent and another due to stone formation causing retention (Table 9).

In our series, we did not observe any other stricture recurrence or urinary retention. We also did not observe any urinary tract infections.

## Discussion

This retrospective review is one of the few studies of the Memokath^®^045 stent in the literature, especially for the treatment of VUAS post prostatectomy. The limitations of this study include the retrospective nature, the limited number of patients involved and the unknown long-term follow-up results.

The surgical technique has a short operating time, it is easy to learn, and patients can go home the same day. The use of the Memokath^®^045 stent resulted in improved IPSS scores, quality of life and flow rate. Continence results showed that 93% of our patients achieved full continence after a minimum 12-month follow-up, whilst the remaining 7% achieved social continence. 21% of our patients encountered minor complications (Clavien grade 1 or 2), which were successfully managed conservatively. 10% of patients required a re-operation due to inadequate stent size or stent migration. Additionally, in 7%, the stent failed and was removed and not replaced due to re-stricture and stone formation.

Overall, the use of the Memokath^®^045 stent for the management of VUAS in patients who have previously failed endoscopic dilation is a novel and successful technique. It is less invasive than other techniques such as bladder neck reconstruction and urinary diversion and provides superior patency results. Comparative options such as bladder neck reconstruction have been shown to provide successful management in approximately 60–93% of patients. In addition, bladder neck reconstruction is a major complex procedure and has a significant risk of incontinence, resulting in the need for artificial urinary sphincter insertion [8, 9, 13]. Furthermore, urinary diversion is also a major procedure which whilst achieving urinary flow results in worse patient satisfaction outcomes. Comparatively, our series shows that in 93% of patients, the Memokath^®^045 stent was successful with minimal complications and excellent post-operation continence rates.

We would advocate the consideration and utilization of the Memokath^®^045 stent in patients with intractable VUAS following radical prostatectomy.

## Appendix

See appendix Tables 1, 2, 3, 4, 5, 6, 7, 8, 9.

**Table 1:** Number and age of patients

Number of patients	30
Patient age (Median + IQR)	62 (50–73)

**Table 2:** Cause of stricture

Cause of stricture	Number of cases (%)
Radical prostatectomy	20 (67%)
Robot-assisted	16 (53%)
Laparoscopic	3 (10%)
Open	1 (3%)
Radical prostatectomy + adjuvant radiotherapy	4 (13%)
Salvage prostatectomy	6 (20%)
Post radiotherapy	1 (3%)
Post brachytherapy	1 (3%)
Post HIFU	4 (13%)

**Table 3:** Time to insertion

Time from prostatectomy to Memokath^®^045 insertion (months) (mean + SD)	13 (± 3)

**Table 4:** Severity of LUTS prior to Memokath^®^045 insertion

Catheter dependent	20 (67%)
Non-catheter dependent	10 (33%)

**Table 5:** Memokath^®^045 length

5 mm	26 (87%)
10 mm	3 (10%)
20 mm	1 (3%)

**Table 6:** Operation time and length of stay

Stage 1	
Operation time (mins) (mean + SD)	22 (± 6)
Length of stay (DAYS) (mean)	1
Stage 2	
Operation time (MINS) (mean + SD)	12 (± 4)
Length of stay (days) (mean)	1

**Table 7:** Follow-Up Outcomes

	Baseline	3 months post-Memokath^®^045 insertion	3 months post-Memokath^®^045 removal	Minimum 12 months post-Memokath^®^045 removal
IPSS	28 (± 4)	12 (± 3)	12 (± 4)	10 (± 4)
IPSS-QoL	5 (± 1)	2 (± 1)	2 (± 1)	2 (± 1)
*Q*_max_ (mls/s)	6 (± 2)	16.8 (± 4.5)	14.2 (± 6.8)	14 (± 10.2)
PVR (mls)	176 (± 52)	16 (± 6)	20 (± 8)	22 (± 12)
No. of pads used/day (%)				
0		24 (86%)	26 (93%)	26 (93%)
1–2		2 (7%)	2 (7%)	2 (7%)
> 2		2 (7%)	0	0

**Table 8:** Type of complications

Type	Number of patients (%)	Clavien–Dindo	Management
Dysuria	2 (7)	1	Conservative, analgesia
Ejaculatory pain	2 (7)	1	Conservative, analgesia
Temporary urge incontinence	2 (7)	2	Conservative, anticholinergic
Inadequate Memokath^®^045 size	2 (7)	3a	Re-operation and replacement
Memokath^®^045 migration	1 (3)	3a	Re-operation and replacement

**Table 9:** Type of failure—resulting in Memokath^®^045 stent removal

Type	Number of patients (%)
RE-stricture through MEMOKATH^®^045 stent	1 (3)
Stone formation	1 (3)

## References

[1] Elliott SP, Meng MV, Elkin EP (2007). Incidence of urethral stricture after primary treatment for prostate cancer: data from CaPSURE. J Urol.

[2] Breyer BN, Davis CB, Cowan JE, Kane CJ (2010). Carroll PR Incidence of bladder neck contracture after robot-assisted laparoscopic and open radical prostatectomy. BJU Int.

[3] Jarosek SL, Virnig BA, Chu H, Elliott SP (2015). Propensity-weighted Long-term risk of urinary adverse events after prostate cancer surgery, radiation or both. Eur Urol.

[4] Ward JF, Sebo TJ, Blute ML, Zincke H (2005). Salvage surgery for radiorecurrent prostate cancer: contemporary outcomes. J Urol.

[5] Pfalzgraf D, Siegel FP, Kriegmair MC, Wagener N (2017). Bladder neck contracture after radical prostatectomy: what is the reality of care?. J Endourol.

[6] Rocco NR, Zuckerman JM (2017). An update on best practice in the diagnosis and management of post-prostatectomy anastomotic strictures Ther. Adv Urol.

[7] Ishii G, Naruoka T, Kasai K, Hata K, Omono H, Suzuki M, Kimura T, Egawa S (2015). High pressure balloon dilation for vesicourethral anastomotic strictures after radical prostatectomy. BMC Urol.

[8] Sourial MW, Richard PO, Bettez M, Jundi M, Tu LM (2017). Mitomycin-C and urethral dilatation: A safe, effective, and minimally invasive procedure for recurrent vesicourethral anastomotic stenoses. Urol Oncol.

[9] Pfalzgraf D, Beuke M, Isbarn H, Reiss CP, Meyer-Moldenbauer WH, Dahlem R, Fisch M (2011). Open retropubic reanastomosis for highly recurrent and complex bladder neck stenosis. J Urol.

[10] Reiss CP, Pfalzgraf D, Kluth LA (2014). Transperineal reanastomosis for the treatment for highly recurrent anastomotic strictures as a last option before urinary diversion. World J Urol.

[11] Simonato A, Gregori A, Lissiani A (2007). Two-stage transperineal management of posterior urethral stricture or bladder neck contractures associated with urinary incontinence after prostate surgery and endoscopic treatment failures. Eur Urol.

[12] Staios D, Shergill I, Thwaini A, Junaid I, Bucholz N (2007). The Memokath™ stent Expert. Rev Med Devices.

[13] Nikolavsky D, Blakely SA, Hadley DA (2014). Open reconstruction of recurrent vesicourethral anastomotic stricture after radical prostatectomy. Int Urol Nephrol.

